# Comparison of the chondrogenic potential of eBMSCs and eUCMSCs in response to selected peptides and compounds

**DOI:** 10.1186/s12917-024-04448-3

**Published:** 2025-02-17

**Authors:** Boushra Ajeeb, Emi A. Kiyotake, Peggy A. Keefe, Jennifer Nikki Phillips, Jennifer N. Hatzel, Laurie R. Goodrich, Michael S. Detamore

**Affiliations:** 1 Stephenson School of Biomedical Engineering, University of Oklahoma, 101 David L Boren Blvd Norman, Norman, OK 73019 USA; 2 Translational Medicine Institute, Colorado State University, 2350 Gillette Drive, Fort Collins, CO 80521 USA; 3 Department of Clinical Sciences, Colorado State University, Fort Collins, CO USA; 4 Department of Biomedical Sciences, Colorado State University, 3101 Rampart Road, Fort Collins, CO 80521 USA

**Keywords:** Cartilage regeneration, Chondroinductive peptides, Chondrogenesis

## Abstract

**Background:**

Cartilage injuries pose significant challenges in horses and often lead to post-traumatic osteoarthritis (PTOA). Despite the advances in surgical and regenerative techniques, the result in most cases is the formation of a fibrocartilage repair tissue. Cell-based cartilage therapies are mainly focused on equine bone marrow-derived mesenchymal stem cells (eBMSCs) as they are easily accessible, and multipotent. Nonetheless, alternative allogeneic sources, for example equine umbilical cord matrix mesenchymal stromal cells (eUCMSCs), hold promise given their non-invasive and readily accessible nature. Considerable research has been dedicated to exploring chondroinductive factors (e.g., peptides and small compounds), aiming to replace growth factors for inducing chondrogenesis. However, these factors have not yet translated to the equine community. Therefore, in the current study, we selected from the literature two promising peptides, CM10 and CK2.1, and two promising compounds, kartogenin and SM04690, and assessed their chondroinductive potential with both eBMSCs and eUCMSCs. In addition, the chondroinductive potential of eBMSCs was evaluated in monolayer and spheroid culture in both hypoxia and normoxia in response to dexamethasone and/or transforming growth factor beta 3 (TGF-β3).

**Results:**

Following 21 days of culture, none of the evaluated chondrogenic factors resulted in a higher gene expression of chondrogenic markers compared to the positive or negative controls with eBMSCs or eUCMSCs. Interestingly, spheroid culture in hypoxia with dexamethasone treatment (without TGF-β or any compound or peptide) was sufficient to induce the chondrogenic differentiation of eBMSCs.

**Conclusion:**

Based on cell response to the positive control, in the conditions employed in the current study, eBMSCs may be preferred over eUCMSCs for chondrogenesis. The current study supports the use of spheroid culture, and the use of dexamethasone over TGF-β or any of the compounds or peptides tested here from the prior literature to drive chondrogenesis with eBMSCs.

## Background

Equine musculoskeletal diseases are the most common debilitating diseases in horses, with osteoarthritis representing the most prevalent condition [1, 2]. Currently used treatments include anti-inflammatory drugs, which are mainly nonsteroidal anti-inflammatory drugs (NSAIDS) [3, 4] (intravenous) or corticosteroids (intraarticular) [4]. Other treatments include intraarticular injections of hyaluronic acid or polysulfated polysaccharides [4]. Regenerative therapies have become more common in the past few decades and are referred to as orthobiologics [4, 5], and include autologous-conditioned serum [6, 7], autologous-protein solution [8–10], platelet-rich plasma [11–13], and mesenchymal stem cells (MSCs) [14–17]. Surgical interventions [4, 18–20] include bone-marrow stimulation [21], autologous chondrocyte implantation (ACI) [22], and osteochondral grafting [23]. In many cases, the combination of surgical techniques and intraarticular therapies provide a better outcome [4].

In cell therapy, autologous equine bone marrow-derived stem cells (eBMSCs) are the primary cell type used for the treatment of equine musculoskeletal diseases in horses. However, in the past decade, the use of *allogeneic* eBMSCs increased because these allogeneic cells offer the value of an off-the-shelf therapy [15]. Given the limited supply of allogeneic eBMSCs that have been pre-screened for potential efficacy, there has been an interest in investigating the chondroinductive potential of other allogeneic cell sources, of which umbilical cord blood mesenchymal stem cells (UCB-MSCs) and umbilical cord matrix mesenchymal stromal cells (UCMSCs) represent attractive options that are readily available, do not require invasive procedures to collect, and have possible immunomodulatory potential [24–26]. While most of the literature on equine umbilical cord cells focuses on eUCB-MSCs, a few studies have evaluated the chondroinductive potential of eUCMSCs. For example, in 2011, Lovati et al. [26] compared the chondroinductive potential of eUCMSCs, equine amniotic fluid mesenchymal stem cells (eAF-MSCs), or eBMSCs in pellet culture (500,000 cells/pellet) in response to TGF-β1, and found that eUCMSCs showed limited chondroinductive potential compared to the other cell sources based on histological staining and biochemical assays. In a more recent (2018) example, Rakic et al. [25] evaluated the chondroinductive potential of eUCMSCs and eUCB-MSCs in response to BMP-2 with TGF-β1 in 3D on a collagen scaffold and found that eUCMSCs had a limited chondroinductive potential as compared to eUCB-MSCs. Our previous work with human UCMSCs demonstrated some potential for chondrogenesis [27–29], providing motivation to revisit UCMSCs from an equine source with new chondroinductive signals.

Peptides and small compounds have recently gained attention for cartilage regeneration [30, 31]. Chondroinductive compounds could be used to enhance cell therapy or to precondition MSCs prior to intraarticular injection. Among the evaluated compounds in the literature, kartogenin (KGN) [32–34] and SM04690 [35, 36] have shown some promise for chondrogenic differentiation of BMSCs. As for peptides, CM10 [37–39] and CK2.1 [40–42] appear to be promising candidates for the chondrogenic differentiation of BMSCs [30]. A previous study from our team showed no evidence of chondroinduction with human BMSCs [43] with kartogenin, SM04690, CM10, or CK2.1; however, as of February 2024, there are no published studies that have evaluated the chondroinductive potential of the aforementioned peptides or compounds with equine MSCs.

Hypoxia has been reported to enhance the chondrogenic differentiation of human BMSCs [44, 45]. With equine MSCs, only one study evaluated eBMSCs in hypoxia macropellets (500,000 cells/pellet) [46] and another study evaluated eUCMSCs in hypoxia in a collagen scaffold [25]. Both studies found hypoxia and normoxia to be equivalent in terms of the gene expression of chondrogenic markers. More studies evaluating hypoxia versus normoxia for chondrogenesis with equine cells are needed, especially given that the native cartilage environment is under hypoxia, therefore, we evaluated both conditions in this study.

In the current study, we evaluated and compared the chondroinductive potential of kartogenin, SM04690, CM10, and CK2.1 to a TGF-β3 positive control with eUCMSCs and eBMSCs in hypoxia in spheroid culture. Additionally, knowing that BMSCs from different species respond differently to culture conditions and growth factors, we compared the chondroinductive potential of eBMSCs in monolayer and spheroid culture, in response to dexamethasone with or without TGF-β3 in both hypoxia and normoxia. Our hypotheses were that eUCMSCs would have chondroinductive potential comparable to eBMSCs, and that at least one of the peptides or compounds would induce the chondrogenic differentiation of eBMSCs and eUCMSCs. Additionally, we hypothesized that dexamethasone would induce the chondrogenic differentiation of eBMSCs in the absence of TGF-β3.

## Results

### Flow cytometry

**Fig. 1:**
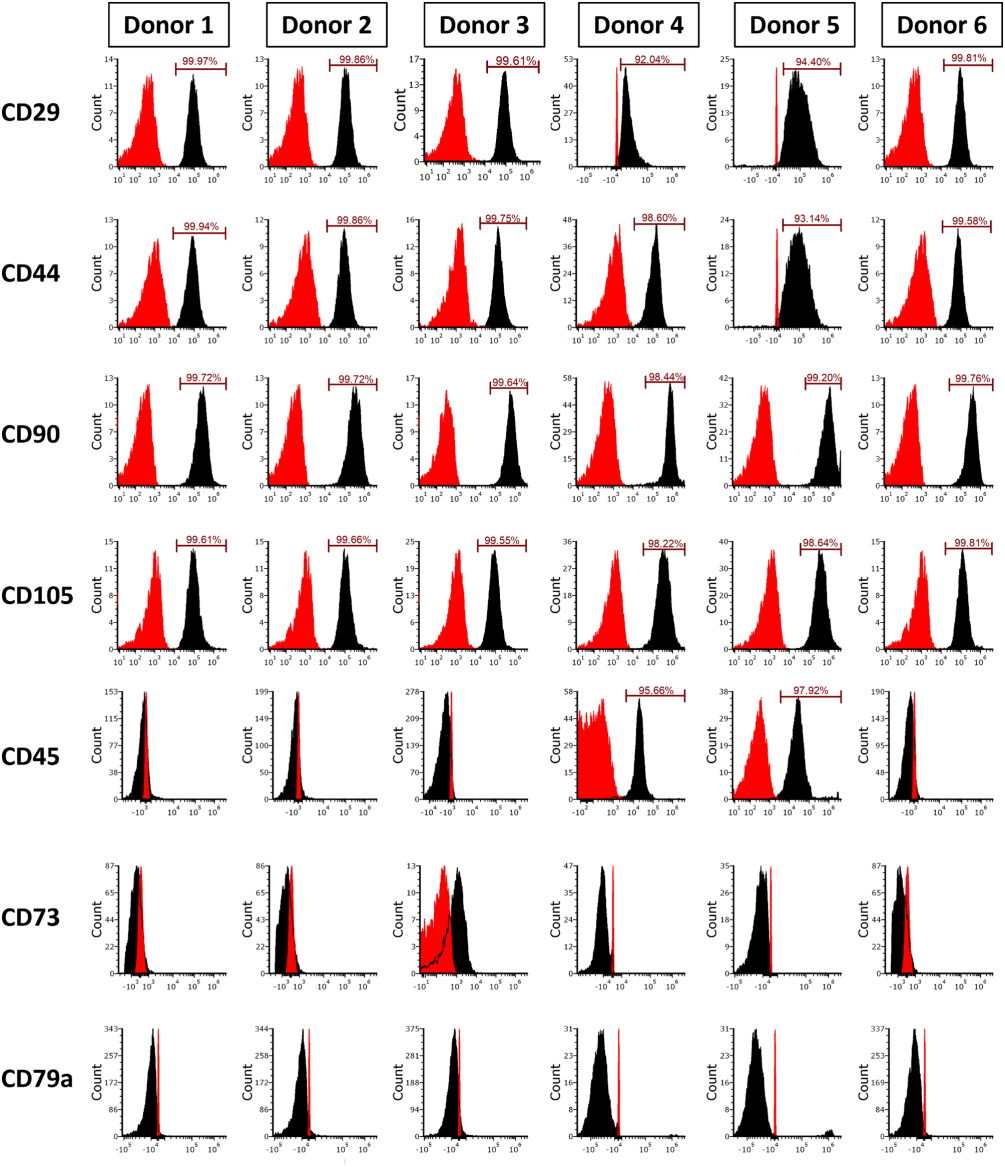
Flow cytometric histogram analyses of cell surface marker expressions of eBMSCs at Passage 4: All eBMSC donors expressed CD29, CD44, CD90, and CD105 while being negative for CD73 and CD79a. Donors 1, 2, 3, and 6 were negative for CD45, whereas donors 4, and 5 were slightly positive for CD45. Red histograms represent unstained controls and black histogram represent stained samples. eBMSCs: Equine bone marrow-derived bone marrow-derived mesenchymal stem cells

Characterization of passage 4 eBMSCs and eUCMSCs was performed using flow cytometric analysis of cell surface markers CD29, CD44, CD45, CD73, CD79a, CD90, and CD105. For eBMSCs (Fig. 1), all six donors were positive for CD29, CD44, CD90, and CD105. In addition, all six eBMSC donors were negative for CD73 and CD79a. While donors 1, 2, 3, and 6 were negative for the hematopoietic cell marker CD45, donors 4 and 5 surprisingly exhibited positive CD45 staining.

**Fig. 2:**
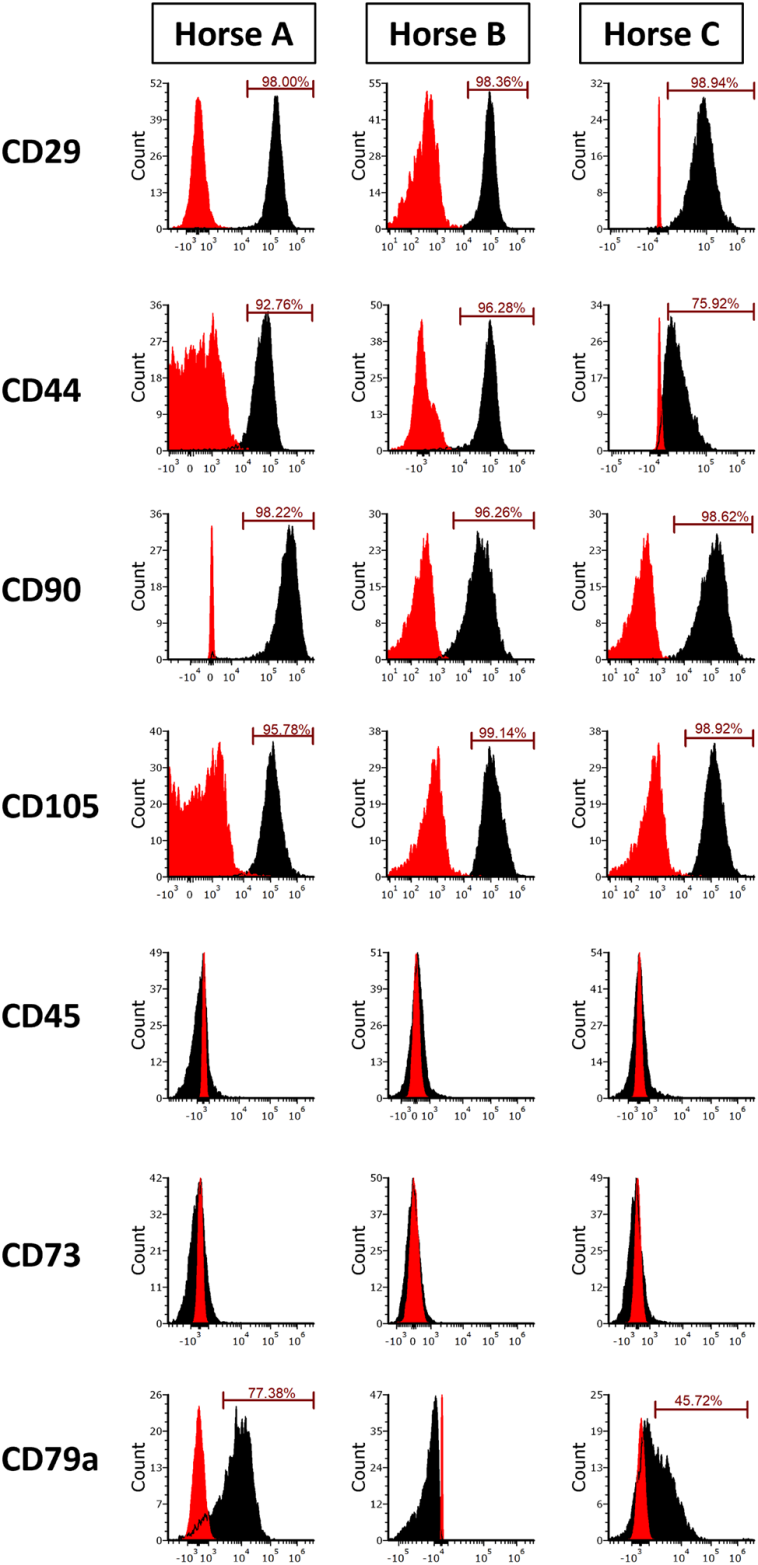
Flow cytometric histogram analyses of cell surface marker expressions of (eUCMSCs) at Passage 4: All eUCMSC donors expressed CD29, CD44, CD90, and CD105, while being negative for CD45, and CD73. Donors 1, and 3 were slightly positive for CD79A. Red histograms represent unstained controls and black histogram represent stained samples. eUCMSCs: Equine umbilical cord-derived mesenchymal stromal cells

For eUCMSCs (Fig. 2), cells harvested from the three different umbilical cords were positive for CD29 at a percentage greater than 98%. Donors 1, 2, and 3 were 92.8%, 96.3%, and 75.9% positive for CD44, respectively. For CD90 and CD105, more than 95% of the cells from all donors were positive. All three donors were negative for CD73 and CD45, whereas for CD79a, variable expression was observed among donors. Specifically, donor 2 was negative for CD79a, whereas donor 1 was 77.4% positive for CD79a and donor 3 was 45.7% positive for CD79a.

### Gene expression

**Fig. 3:**
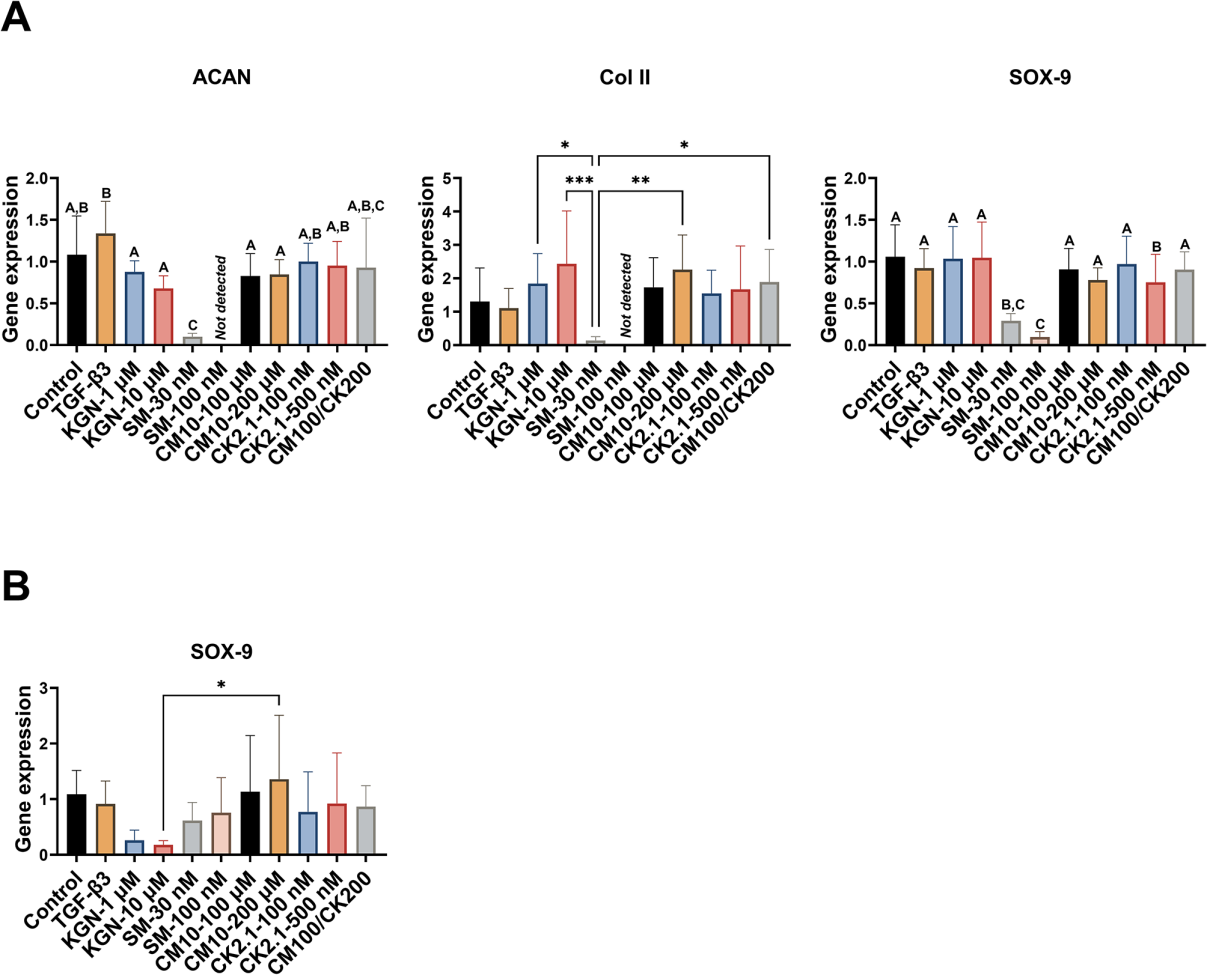
Gene expression after 21 days in spheroid culture under hypoxic conditions (5% O_2_). **A**) Equine bone marrow-derived mesenchymal stem cells (eBMSCs): no compound or peptide significantly increased chondrogenic gene expression of SOX-9, ACAN, or Col2a1 in eBMSCs compared to the control group. Interestingly, SM-30 nM and SM-100 nM *decreased* the gene expression of all three genes compared to the control and certain other groups. **B**) Equine umbilical cord-derived mesenchymal stromal cells (eUCMSCs): the eUCMSCs treated with CM10-200 µM had 7.6 times greater SOX-9 gene expression than that of the eUCMSCs treated with KGN-10 µM. No other differences were significant. There was no detectable ACAN gene expression except in some of the TGF-β3 samples (data not shown), and there was no detectable col2a1 gene expression in any of the samples. * *p* < 0.05, ** *p* < 0.01, *** *p* < 0.001, each letter indicated significance from groups with other letters. *n* = 8 for Reported values are mean + standard deviation. SM = SM04690, KGN = Kartogenin, CM100/CK200 = CM10 at 200 µM and CK2.1 and 200 nM, ACAN = Aggrecan, col II = collagen type II (subtype col2a1), col I = collagen type I

The gene expression of eBMSCs and eUCMSCs in response to kartogenin, SM04690, CM10, and CK2.1 are shown in Fig. 3. Starting with eBMSCs (Fig. 3A), no compound or peptide significantly increased chondrogenic gene expression of SOX-9, ACAN, or Col2a1 in eBMSCs compared to the negative control group. Interestingly, the TGF-β3 positive control did not induce any significantly higher gene expression of ACAN, Col2a1, or SOX-9 compared to the negative control group (noting that dexamethasone was present in both the negative and positive control media). SM04690 at 30 nM had 90% lower ACAN gene expression than the negative control (*p* < 0.0001), and no ACAN was detected for SM04690 at 100 nM. Additionally, the combination of CM10 and CK2.1 did not induce any significant gene change in ACAN gene expression compared to the negative control. For Col2a1, no gene expression was detected with SM04690 at 100 nM. SM04690 had the lowest detectable Col2a1 gene expression at 30 nM; however, the lower value was not significantly different from the negative control, but was 92.4% lower than KGN-1 µM (*p* < 0.05), 94.2% lower than KGN-10 µM (*p* < 0.0001), 93.8% lower than CM10-200 µM (*p* < 0.01), and 93.0% lower than CM10/CK2.1 (*p* < 0.05). As for SOX-9, SM04690 at 100 nM resulted in the lowest gene SOX-9 gene expression including a 91% lower value (*p* < 0.0001) compared to the negative control. Interestingly, no collagen I gene expression was observed in any of the groups.

For eUCMSCs (Fig. 3B), only SOX-9 gene expression was detected and there were no significant differences in SOX-9 expression among groups except for CM10-200 µM having an 87% lower value (*p* < 0.05) compared to KGN-10 µM. No collagen I gene expression was observed with any of the groups.

**Fig. 4:**
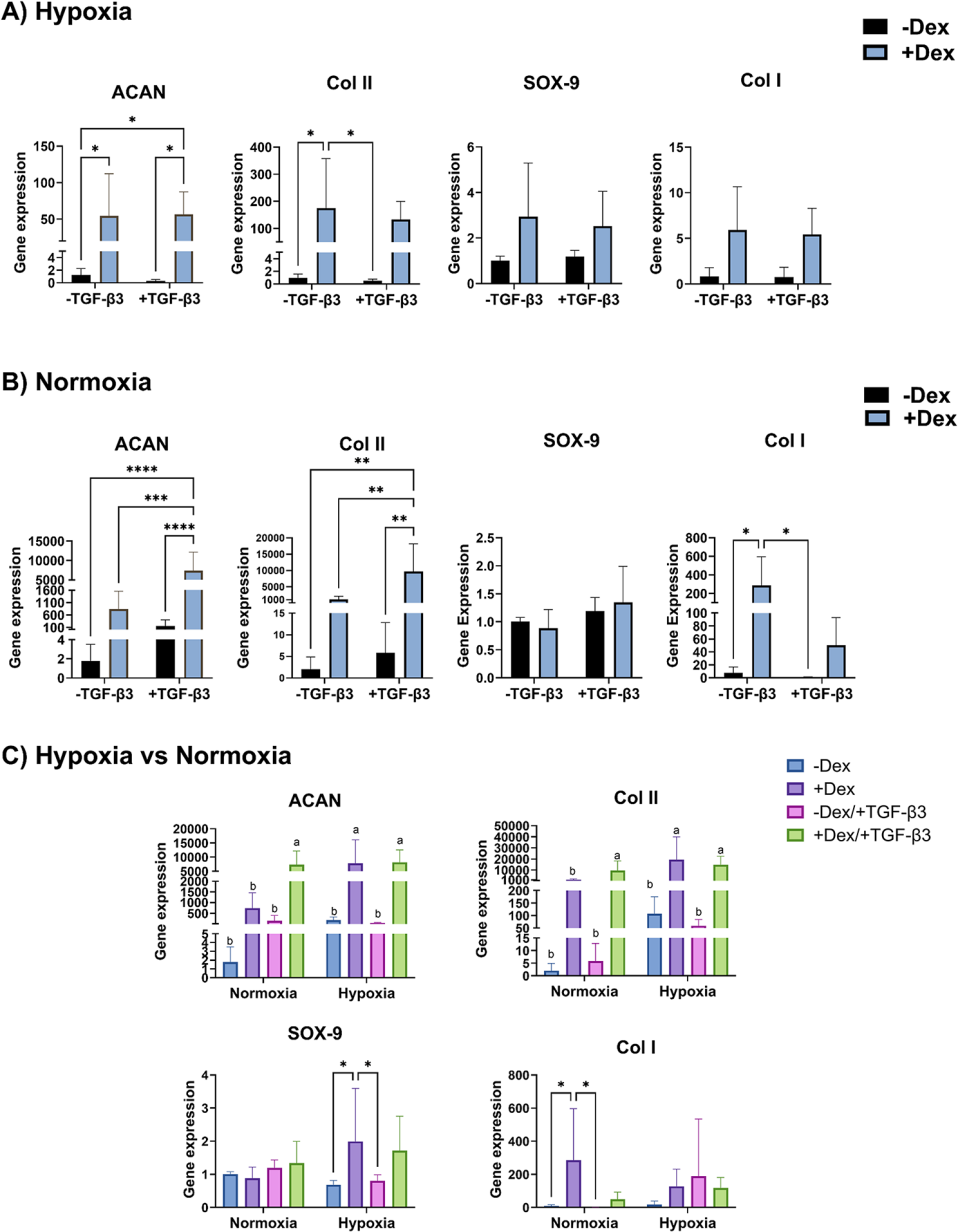
Gene expression of eBMSCs in response to dexamethasone in hypoxia or normoxia in spheroid culture. **A**) In hypoxic conditions, after 21 days, eBMSCs treated with dexamethasone had 43-fold and 181-fold higher ACAN and col2a1 gene expressions, respectively, compared to the negative control (i.e., no dexamethasone and no TGF-β3), whereas eBMSCs treated with both dexamethasone and TGF-β3 had 45-fold and 138-fold higher ACAN and Col2a1 gene expressions, respectively, compared to the negative control. No significant differences among groups were observed for SOX-9 or collagen I gene expression. **B**) In normoxia, after 21 days, eBMSCs treated with both dexamethasone and TGF-β3 had the highest gene expression for ACAN and col2a1, with 4108-fold and 4705-fold higher gene expressions, respectively, compared to the negative control. No significant differences were observed among groups for SOX-9. As for collagen I, eBMSCs treated with dexamethasone alone exhibited the highest gene expression with 38-fold higher gene expression compared to the negative control. **C**) Normalizing the gene expression to the normoxia control group, in hypoxic conditions, eBMSCs treated with only dexamethasone resulted in 90.5% and 94.3% higher ACAN and Col2a1 gene expression, respectively, compared to eBMSCs treated with only dexamethasone in normoxia; For SOX-9 and collagen I, no significant differences were observed between hypoxia and normoxia for the remaining groups. * *p* < 0.05, ** *p* < 0.01, *** *p* < 0.001, *n* = 6–8. Reported values are mean + standard deviation. eBMSCs: Equine bone marrow-derived bone marrow-derived mesenchymal stem cells, ACAN = Aggrecan, col II = collagen type II (subtype col2a1), col I = collagen type I

Due to the similar eBMSC responses between the negative and positive controls (Fig. 3A), as a next step we transitioned to evaluate the effect of dexamethasone, with and without TGF-β3, with eBMSCs. In eBMSC spheroid culture in hypoxia (Fig. 4A), for all genes there were no significant differences between eBMSC spheroids treated with dexamethasone with or without TGF-β3, consistent with the previous experiment (i.e., the positive vs. negative controls). Likewise, in the absence of dexamethasone, no significant differences were observed between eBMSC spheroids treated with or without TGF-β3 for all genes. In contrast to TGF-β3, which had no significant effect in hypoxia, dexamethasone led to some increases of an order of magnitude or two for chondrogenic genes.

Specifically, and interestingly, large differences for eBMSC spheroids in hypoxia were observed compared to the negative control (i.e., no dexamethasone and no TGF-β3) for ACAN and Col2a1. For example, eBMSC spheroids treated with dexamethasone alone had 43-fold (*p* < 0.05) and 181-fold higher (*p* < 0.05) ACAN and Col2a1 gene expressions, respectively, compared to the negative control (Fig. 4A). eBMSCs treated with both dexamethasone and TGF-β3 had 45-fold (*p* < 0.05) higher ACAN gene expression compared to the negative control, but not a significantly different gene expression for Col2a1 gene expression. In the presence of TGF-β3, eBMSC spheroids treated with dexamethasone had 185-fold higher (*p* < 0.05) ACAN gene expression compared to eBMSC spheroids without dexamethasone; however, no significant differences were observed for the remaining genes. No significant differences were observed among groups for either collagen I or SOX-9 in hypoxia (Fig. 4A).

In normoxia (Fig. 4B), the eBMSC spheroids treated with both dexamethasone and TGF-β3 had the highest gene expression for ACAN and Col2a1, with three orders of magnitude, i.e., 4,108-fold (*p* < 0.0001) and 4,705-fold (*p* < 0.01) higher gene expressions, respectively, compared to the negative control. For eBMSC spheroids treated with dexamethasone alone, despite a mean value that was 412-fold higher for ACAN and 539-fold higher for Col2a1 compared to the negative control, the difference was not statistically significant. In the absence of dexamethasone, no significant differences were observed between eBMSC spheroids treated with or without TGF-β3 for all genes, similar to what was observed in hypoxia. However, in the presence of dexamethasone, eBMSC spheroids treated with TGF-β3 had 10-fold higher (*p* < 0.001) and 9-fold higher (*p* < 0.01) ACAN and Col2a1 gene expression, respectively, compared to eBMSCs treated with dexamethasone alone. In the presence of TGF-β3, eBMSC spheroids treated with dexamethasone had 48-fold (*p* < 0.0001) and 2,136-fold higher (*p* < 0.01) gene expression of ACAN and Col2a1, respectively, compared to eBMSC spheroids treated without dexamethasone. No significant differences were observed among groups for SOX-9. As for collagen I, eBMSC spheroids treated with dexamethasone alone exhibited the highest gene expression with a 38-fold higher (*p* < 0.05) gene expression compared to the negative control and 448-fold higher (*p* < 0.05) gene expression compared to eBMSC spheroids treated with TGF-β3 alone. No other significant differences were observed among the remaining groups for collagen I gene expression.

**Fig. 5:**
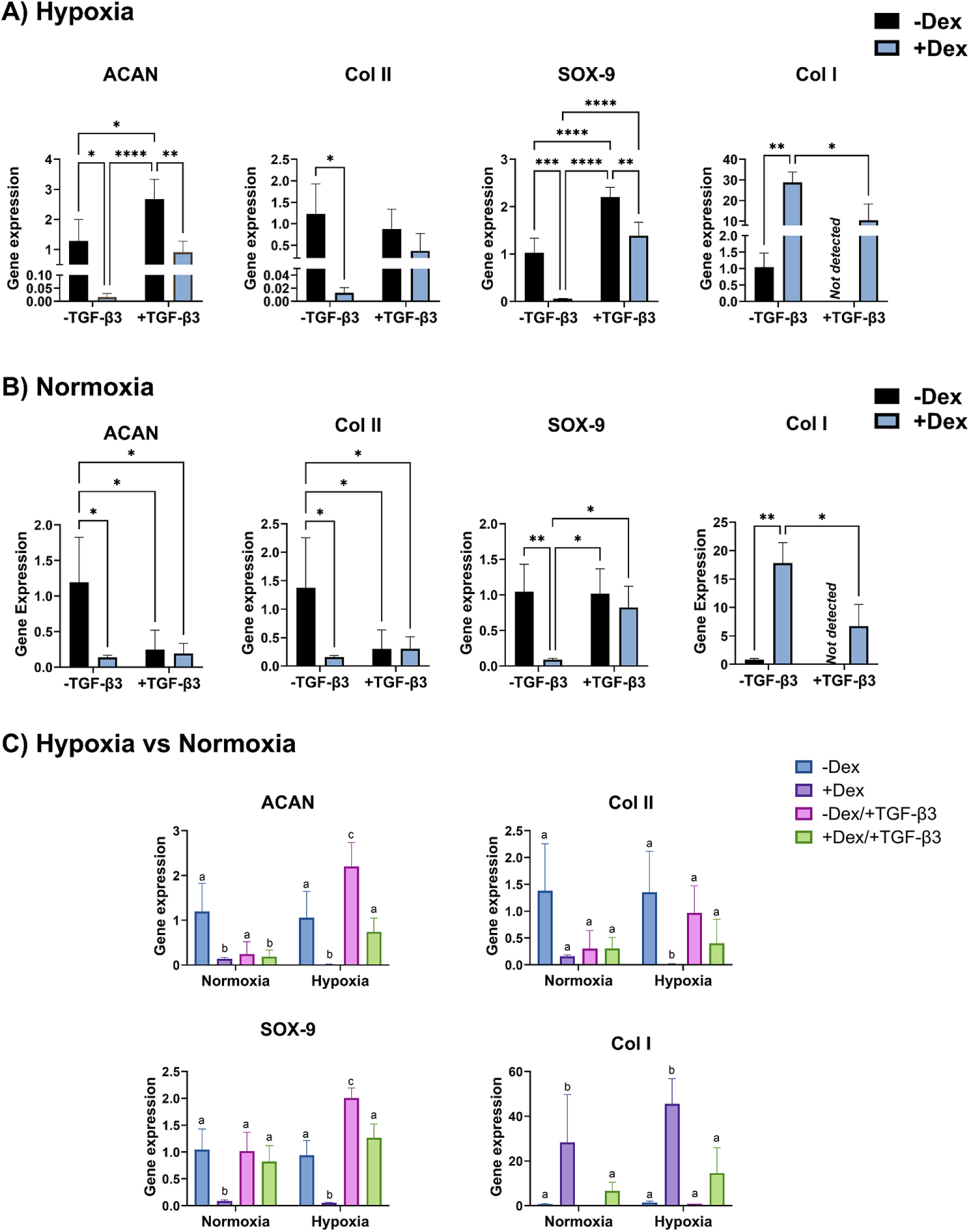
Gene expression of eBMSCs in response to dexamethasone in hypoxia or normoxia in monolayer culture. **A**) In hypoxic conditions, after 21 days, eBMSCs treated with only dexamethasone resulted in significantly lower ACAN, col2a1, and SOX-9 gene expression as compared to the negative control; however, collagen I expression was 27-fold higher compared to the negative control. Treatment with TGF-β3 alone had 2-fold higher ACAN and SOX-9 gene expression, compared to the negative control. Treatment with both dexamethasone and TGF-β3 did not result in a significant change in the gene expression of ACAN, col2a1, SOX-9, or Col I. **B**) In normoxia, after 21 days, eBMSCs treated with dexamethasone, TGF-β3, or both resulted in significantly lower gene expression of ACAN and col2a1. Treatment with dexamethasone alone resulted in significantly lower SOX-9 gene expression and the highest gene expression of collagen I compared to the negative control. **C**) Normalizing the gene expression to the normoxia control group, in hypoxic conditions, after 21 days, eBMSCs treated with only TGF-β3 resulted in 89% and 49% higher ACAN and SOX-9 gene expression, respectively compared to eBMSCs treated with only TGF-β3 in normoxia. * *p* < 0.05, ** *p* < 0.01, *** *p* < 0.001, *n* = 3–4. Reported values are mean + standard deviation. eBMSCs **=** Equine bone marrow-derived bone marrow-derived mesenchymal stem cells, ACAN = Aggrecan, col II = collagen type II (subtype col2a1), col I = collagen type I

The responses of eBMSCs in monolayer culture differed from those in spheroid culture. For the gene expression of eBMSCs in monolayer culture, in hypoxia (Fig. 5A), eBMSCs treated with TGF-β3 alone resulted in the highest gene expression of ACAN and SOX-9, including 2.0-fold higher (*p* < 0.05) and 2.1-fold higher (*p* < 0.0001) values, respectively, compared to the negative control. In the presence of dexamethasone, eBMSCs treated with TGF-β3 had 23-fold (*p* < 0.0001) higher SOX-9 gene expression compared to treatment with dexamethasone alone. For eBMSCs treated with dexamethasone alone, 2.8-fold higher (*p* < 0.05) collagen I gene expression was observed, compared to eBMSCs treated with both dexamethasone and TGF-β3, and 27.4-fold higher (*p* < 0.01) value compared to the negative control. In the absence of TGF-β3, eBMSCs treated with dexamethasone had 98.8% (*p* < 0.05), 98.9% (*p* < 0.05), and 94.1% (*p* < 0.001) lower expressions of ACAN, Col2a1, and SOX-9, respectively. In the presence of TGF-β3, eBMSCs without dexamethasone treatment had 3-fold (*p* < 0.01) and 1.6-fold (*p* < 0.01) higher ACAN and SOX-9 gene expressions compared to eBMSCs treated with dexamethasone; however, no significant differences were observed for Col2a1 or collagen I gene expression.

In normoxia (Fig. 5B), in the absence of TGF-β3, eBMSCs treated *without* dexamethasone resulted in 8.6-fold (*p* < 0.05), 8.8-fold (*p* < 0.05), and 11.7-fold (*p* < 0.01) higher gene expression of ACAN, Col2a1, and SOX-9, respectively, compared to treatment with dexamethasone. Again, in the absence of TGF-β3, eBMSC collagen I expression in contrast increased by 22.4-fold (*p* > 0.01) with dexamethasone compared to without dexamethasone. In the presence of TGF-β3 in normoxia, no significant differences were observed between eBMSCs treated with or without dexamethasone for all genes. In the presence of dexamethasone, no significant differences were observed between eBMSCs treated with or without TGF-β3 for ACAN and Col2a1 gene expression; however, 9-fold (*p* < 0.05) higher SOX-9 and 62.2% (*p* < 0.05) lower collagen I gene expressions were observed with eBMSCs treated with TGF-β3. Again, in the absence of dexamethasone in normoxia, no significant difference was observed with the addition of TGF-β3 for SOX-9, and no collagen I was detected for eBMSCs treated with TGF-β3 alone.

Normalizing the gene expression to the normoxia control group to compare hypoxia and normoxia (Figs. 4C and 5C). In spheroid culture (Fig. 4C), eBMSCs treated with only dexamethasone in hypoxia resulted in 90.5% and 94.3% higher ACAN and Col2a1 gene expression, respectively, compared to eBMSCs treated with only dexamethasone in normoxia. For both aggrecan and Col2a1 expression, there were three groups that were significantly higher than all other groups, but not significantly different from each other: the TGF-β3 with dexamethasone groups in both normoxia and hypoxia, and the dexamethasone group in hypoxia. However, no significant differences were observed between normoxia and hypoxia for groups treated with TGF-β3 alone or both dexamethasone and TGF-β3. For SOX-9 and collagen I, no significant differences were observed between hypoxia and normoxia. In monolayer culture (Fig. 5C), eBMSCs treated with TGF-β3 alone in hypoxia resulted in 8.9-fold and 2-fold higher ACAN and SOX-9 gene expression, respectively, compared to eBMSCs treated with only TGF-β3 in normoxia. For eBMSCs treated with both dexamethasone and TGF-β3 in hypoxia a 3.9-fold higher ACAN gene expression was observed compared to eBMSCs treated with both dexamethasone and TGF-β3 in normoxia. Treatment of eBMSCs with dexamethasone alone in hypoxia resulted in 10.9-fold lower Col2a1 gene expression compared to eBMSCs treated with dexamethasone alone in normoxia. No other significant differences were observed between hypoxia and normoxia for the remaining groups.

### Biochemical assays

**Fig. 6:**
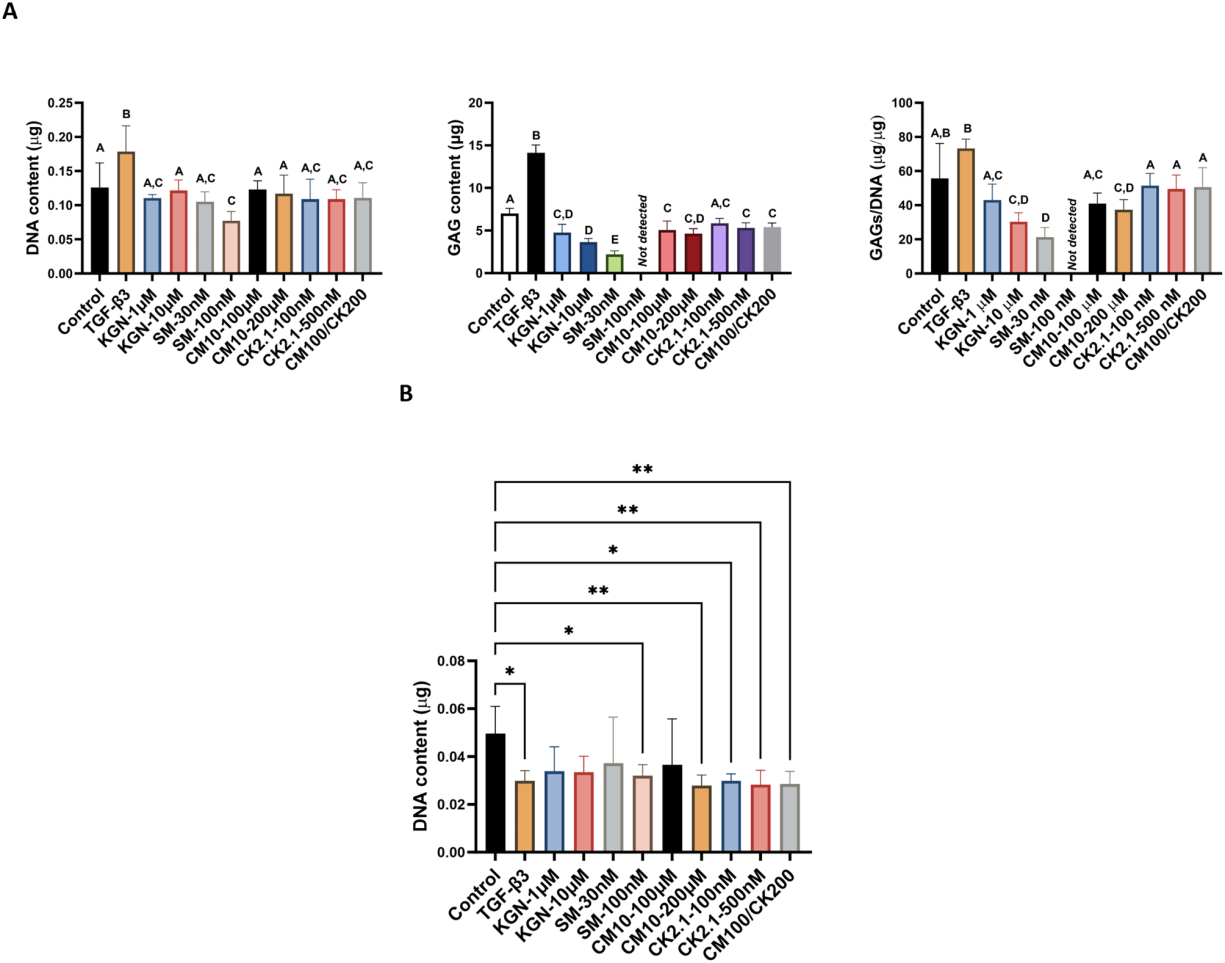
Glycosaminoglycan (GAG) synthesis after 21 days in spheroid culture. **A**) Equine bone marrow-derived mesenchymal stem cells (eBMSCs): eBMSCs treated with TGF-β3 had the highest GAGs/DNA production with a 30% increase compared to the negative control. SM-30 nM had the lowest GAGs/DNA production, and no GAGs were detected with SM-100 nM. **B**) Equine umbilical cord-derived mesenchymal stromal cells (eUCMSCs): For eUCMSCs, GAG was not detected in any of the samples. * *p* < 0.05, ** *p* < 0.01, *n* = 4–8. Reported values are mean + standard deviation. SM = SM04690, KGN = Kartogenin, CM100/CK200 = CM10 at 200 µM and CK2.1 at 200 nM

The glycosaminoglycan (GAG) contents of eBMSCs and eUCMSCs in response to kartogenin, SM04690, CM10, and CK2.1 are shown in Fig. 6. For eBMSCs (Fig. 6A), following 21 days in spheroid culture, the TGF-β3 positive control had the highest DNA content including a 41.7% higher (*p* < 0.001) value compared to the negative control. SM04690 at 100 nM had 38.7% (*p* < 0.01) lower DNA content compared to the negative control. For GAG content, the TGF-β3 group had the highest GAG content including a 2.0-fold higher value (*p* < 0.0001) compared to the negative control. Almost every other group produced less GAG than the negative control. The SM04690 groups had the lowest GAG content among all groups; SM04690 at 30 nM had 68.5% lower (*p* < 0.0001) GAG content than the negative control, and no GAG content was detected in the SM04690 group at 100 nM. KGN at 1 µM and at 10 µM had 32% lower (*p* < 0.0001) and 48% lower (*p* < 0.0001) GAG contents, respectively, compared to the negative control. CM10 at 100µM and at 200 µM had 27% (*p* < 0.0001) and 34% (*p* < 0.0001) lower GAG contents, respectively, compared to the negative control. CK2.1 at 500 nM and CM10/CK2.1 had 24% (*p* < 0.001) and 23% (*p* < 0.01) lower GAG contents compared to the negative control, respectively. As for the GAG content normalized to DNA content, no significant difference was observed between the negative and positive control groups. The SM04690 groups produced significantly less GAG/DNA than all but one of the other groups; most notably SM04690 at 30 nM had 62% (*p* < 0.0001) less GAG/DNA compared to the negative control. KGN at 10 µM and CM10 at 200 µM had 46% (*p* < 0.001) and 33% (*p* < 0.05) lower GAG/DNA compared to the negative control. No significant differences in GAG/DNA content were observed between the negative control and the remaining experimental groups.

For eUCMSCs (Fig. 6B), GAGs were not detected in any of the groups. Therefore, only DNA content was reported, with the negative control exhibiting the highest DNA content, which was significant compared to six groups, including 66.0% higher DNA content than the positive control (*p* < 0.05).

**Fig. 7:**
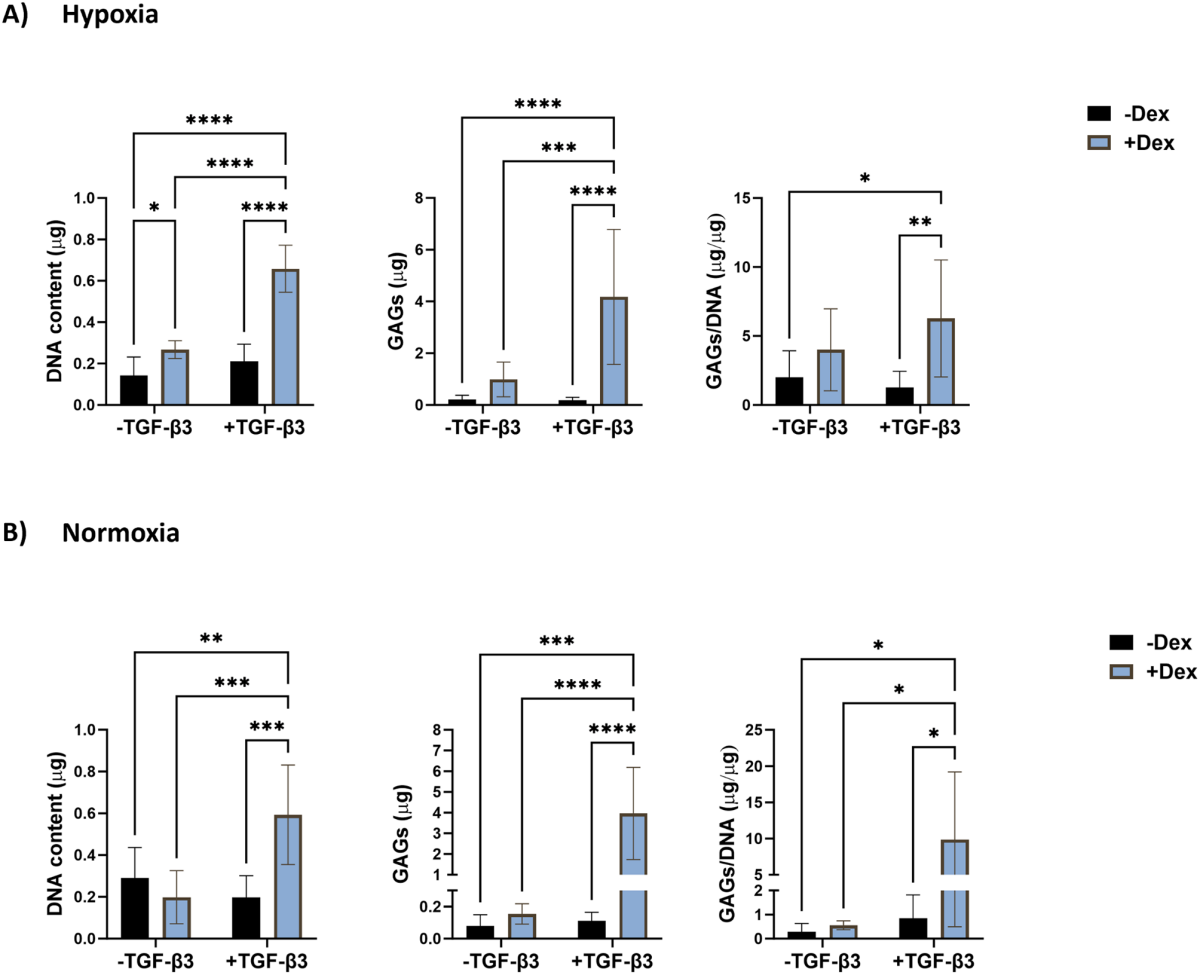
Glycosaminoglycan synthesis of eBMSCs in response to dexamethasone in hypoxia or normoxia in spheroid culture. After 21 days in culture, **A**) In hypoxic conditions, eBMSCs treated with both dexamethasone and TGF-β3 resulted in 3-fold higher GAG/DNA content compared to the negative control. **B**) In normoxia, after 21 days, eBMSCs treated with both dexamethasone and TGF-β3 resulted in 35-fold higher GAG/DNA production compared to the negative control. * *p* < 0.05, ** *p* < 0.01, *** *p* < 0.001, *n* = 6–8. Reported values are mean ± standard deviation. eBMSCs: Equine bone marrow-derived bone marrow-derived mesenchymal stem cells

For the evaluation of the effect of dexamethasone on the GAG production in eBMSC spheroids (Fig. 7), in hypoxia, in the absence of TGF-β3, treatment with dexamethasone resulted in 1.9-fold higher (*p* < 0.05) DNA content compared to the negative control, with no significant differences observed for GAG and GAG/DNA content compared to the negative control. In the presence of TGF-β3, eBMSCs treated with dexamethasone had significantly higher DNA content (3.1-fold (*p* < 0.0001)), GAG content (21.6-fold (*p* < 0.0001)), and GAG/DNA content (4.9-fold (*p* < 0.01)) compared to eBMSCs not treated with dexamethasone. Additionally, treatment with both dexamethasone and TGF-β3 had significantly higher DNA content (4.6-fold (*p* < 0.0001)), GAG content (19.6-fold (*p* < 0.0001)), and GAG/DNA content (3-fold (*p* < 0.05)) compared to the negative control (i.e., no dexamethasone and no TGF-β3). Treatment of eBMSCs with TGF-β3 alone did not result in any significant differences for DNA, GAG, or GAG/DNA content compared to the negative control (Fig. 7A). In normoxia (Fig. 7B), in the absence of TGF-β3, treatment with dexamethasone alone did not result in any significant differences in DNA, GAG, or GAG/DNA content. In the presence of TGF-β3, eBMSCs treated with dexamethasone resulted in 3.0-fold (*p* < 0.001), 36.0-fold (*p* < 0.0001), and 11.7-fold (*p* < 0.05) higher DNA content, GAG content, and GAG/DNA content, respectively, compared to eBMSCs not treated with dexamethasone. Additionally, eBMSCs treatment with both dexamethasone and TGF-β3 resulted in 2-fold (*p* < 0.01), 50-fold (*p* < 0.001), and 35-fold (*p* < 0.05) higher DNA content, GAG content, and GAG/DNA content, respectively, compared to the negative control.

**Fig. 8:**
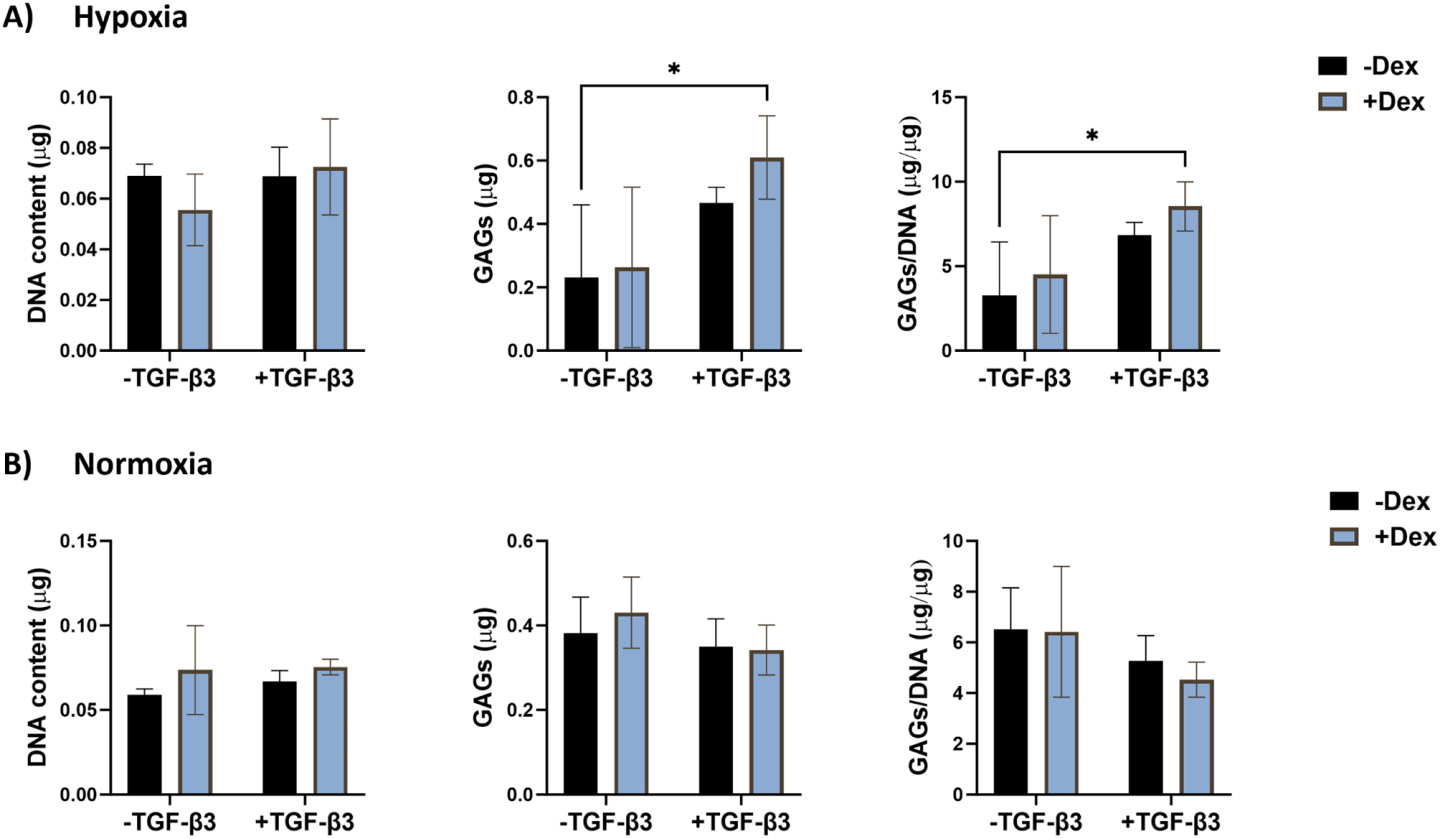
Glycosaminoglycan synthesis of eBMSCs in response to dexamethasone in hypoxia or normoxia in monolayer culture. After 21 days in culture, **A**) In hypoxic conditions, eBMSCs treated with dexamethasone and TGF-β3 resulted in 2.6-fold higher GAG production normalized to DNA content compared to the negative control. No significant differences were observed among the remaining groups. **B**) In normoxia, no significant differences were observed among the groups. * *p* < 0.05, ** *p* < 0.01, *** *p* < 0.001, *n* = 3–4. Reported values are mean ± standard deviation

In monolayer culture, in hypoxia (Fig. 8A), no significant differences were observed among the experimental groups for DNA content. For GAG and GAG/DNA content, the only significant difference was between the negative control and the treatment with both dexamethasone and TGF-β3, which resulted in 2.6-fold (*p* < 0.05) higher GAG and GAG/DNA contents. In normoxia (Fig. 8B), no significant differences were observed among groups for DNA content, GAG content, or GAG/DNA content.

## Discussion

The current study was the first to evaluate the chondroinductive potential of reported chondroinductive compounds and peptides with eBMSCs or eUCMSCs in vitro, and the first to compare the chondroinductive potential of eBMSCs and eUCMSCs in response to TGF-β3 in pellet culture in hypoxia. Additionally, this is the first study to evaluate and compare the chondroinductive potential of dexamethasone and TGF-β3 in eBMSC spheroid culture, and the first to run the comparison in both normoxia and hypoxia. Our hypotheses addressing the chondroinductive compounds, and the chondroinductive potential of eUCMSCs, were not supported, as there were no chondroinductive compounds that outperformed TGF-β3 in chondrogenesis and there was no evidence to support the chondroinductive potential of eUCMSCs. However, the hypothesis that dexamethasone would induce the chondrogenic differentiation of eBMSCs in the absence of TGF-β3 was supported in spheroid culture in hypoxia.

None of the evaluated peptides (i.e., CM10 and CK2.1) or compounds (i.e., kartogenin and SM04690) induced a higher gene expression of chondrogenic markers (ACAN, Col2a1, and SOX-9) or increased GAGs production compared to the negative control for either eBMSCs or eUCMSCs following 21 days in spheroid culture. The limited efficacy of compounds from the literature with eBMSCs in the current study was in agreement with the results of our recent study that evaluated the chondrogenic potential of the same compounds and peptides with hBMSCs [43]. 

No evidence of chondroinduction was observed with eUCMSCs, as no ACAN or Col2a1 gene expression was detected, and no GAGs were detected following 21 days in culture. The limited chondrogenic potential of eUCMSCs is in agreement with previous studies by Lovati et al. [26]. that reported a similar outcome following the treatment of UCMSCs pellets with TGF-β1 for 15 days in normoxia, and by Rakic et al. [25]. that reported a limited chondroinductive potential of eUCMSCs following BMP-2 + TGF-β1 treatment in both normoxia and hypoxia.

All eBMSC and eUCMSC donors exhibited markers associated with mesenchymal stem cells (i.e., CD29, CD44, CD90, and CD105) in accordance with reported literature [47–49], and consistent with stem cell phenotype. Interestingly, while all eBMSC donors were negative for CD73 and CD79a, donors 4 and 5 exhibited positive CD45 staining. One possible explanation could be that the isolation of BMSCs based on their adherence to plastic is known to result in a heterogenous population of cells, which might include subpopulations of CD45-positive cells of hematopoietic origin, which have been previously reported with rat BMSCs [50]. Other factors that have been reported to influence the expression of CD45 in hBMSCs is the donor age, and culture duration [51] with some reports indicating that most BMSCs derive from CD49a^+^/CD45^med.low^ cells and turn into CD49a^+^/CD45^−^ cells when cultured [51, 52]. Despite being CD45 positive, chondroinductive differentiation was observed with pooled eBMSC donors 4, 5, and 6. As for eUCMSCs, donors 1 and 3 exhibited a CD79a positive phenotype, which might be explained by the heterogeneity of the stromal cell population obtained from the umbilical cord matrix during harvest and culture. As a point of comparison, some references have indicated that MSCs derived from human adipose tissue do exhibit positive CD79a staining [53]. Collectively, the cell surface markers observed in the current study indicated that more rigorous evaluation of stem cell markers for various sources of equine MSCs at different passages is warranted. Additional evaluation of hematopoietic markers such as CD34 and Major Histocompatibility Complex Class II (MHC II) markers might help to further characterize eUCMSCs and eBMSCs in future studies.

The lack of significant differences between the negative and positive controls in our first experiment, for both gene expression and GAG content, warranted additional investigation into the effect of dexamethasone and TGF-β3 on the chondrogenic differentiation of eBMSCs. The current study was the first to evaluate and compare the chondroinductive potential of dexamethasone and TGF-β3 in eBMSC spheroid culture, and that comparison was done in both normoxia and hypoxia. Interestingly, in eBMSC spheroid culture under hypoxia, significantly higher gene expression of ACAN and Col2a1 was observed following treatment with dexamethasone alone compared to the negative control. Regardless of whether hypoxia or normoxia was used, addition of TGF-β3 did not lead to significantly higher chondrogenic gene expression, meaning that dexamethasone in hypoxia was sufficient to induce eBMSC chondrogenesis without TGF-β3. Furthermore, no significant differences were observed in GAG/DNA production between dexamethasone and dexamethasone + TGF-β3 groups, further reiterating that dexamethasone alone might be sufficient to induce the chondrogenic differentiation of eBMSCs in spheroid culture in hypoxia, without the need of additional growth factors.

Noteworthy is that no collagen I was detected in the study comparing the chondroinductive compounds in spheroid culture in hypoxia, whereas we did detect collagen I in the study evaluating the effect of dexamethasone under the same conditions. One possible reason for this discrepancy may be that different donors were used in the aforementioned studies, and two out of the three donors used in the dexamethasone study had positive CD45 staining.

In eBMSC spheroid culture in normoxia, dexamethasone alone did have a higher mean value than the negative control for the gene expressions of ACAN and Col2a1; however, those differences were not significant, which could be due to a type II error (i.e., false negative due to small sample number). The treatment with dexamethasone and TGF-β3 in normoxia resulted in significantly higher ACAN and Col2a1 gene expression and higher GAG/DNA production compared to the negative control and dexamethasone groups, but again did not lead to significantly higher expression than with dexamethasone alone in hypoxia. In other words, a combination of both TGF-β3 and dexamethasone were required to reach the highest aggrecan and Col2a1 expression in normoxia, but in hypoxia that highest expression level was possible with dexamethasone alone.

In the future, it may be worth evaluating markers of hypertrophy (e.g., collagen X) following dexamethasone treatment, as BMSCs can tend to acquire hypertrophic properties during chondrogenic induction.

In monolayer culture of eBMSCs under hypoxia, dexamethasone resulted in a significant decrease in the gene expression of ACAN, Col2a1, and SOX-9, and the addition of TGF-β3 alone resulted in significantly higher gene expression of ACAN, Col2a1, and SOX-9. However, in terms of GAG production, no significant differences were observed among groups. Interestingly, in normoxia in monolayer culture the control group had the highest gene expression for ACAN, Col2a1, and SOX-9. It could be that in eBMSC monolayer culture, dexamethasone induces osteogenesis rather than chondrogenesis, which warrants future evaluation of osteogenic markers (e.g., Runx2, BGLAP). Notably in monolayer culture, in comparing normoxia and hypoxia, eBMSCs treated with both dexamethasone and TGF-β3 in hypoxia resulted in higher ACAN gene expression compared to eBMSCs treated with both dexamethasone and TGF-β3 in normoxia.

The findings of the current study do not support the use of eUCMSCs for cell therapies where chondrogenesis of the cells is the therapeutic goal, as we did not find any evidence of chondroinduction in the current culture conditions. It is noteworthy that eUCMSCs are a heterogenous population of cells and thus may not have the same characteristics or differentiation capacity as a homogenous population of mesenchymal stem cells. However, future work exploring 3D hydrogels with extracellular matrix, infusing fluid flow or mechanical stimulation, or possibly exploring different categories of growth factors might be intriguing. Additionally, there might still be an advantage of using eUCMSCs for their immunomodulatory potential [54–56]. eBMSCs remain the most attractive candidate for cell therapy, and in the current study, dexamethasone in hypoxia was revealed to be a potent and sufficient chondroinductive signal for eBMSC spheroids, without the need for reportedly chondroinductive peptides, compounds, or even TGF-β3. Therefore, in cell therapies where cartilage regeneration in a defect area is the goal, there may be an advantage to employing spheroid culture in hypoxia and priming with dexamethasone to enhance the chondroinductive potential of the eBMSCs. The use of spheroids instead of a cell suspension or monolayer has been shown to offer an advantage with chondrocytes, such as using the chondrospheres [57] (spherox) system, which is a 4th generation ACI that is currently approved for use in Europe. As we look to the future, in vivo evaluation is essential to truly evaluate the advantages of using eBMSC spheroids for cartilage regeneration in equines. Additional in vitro studies may add value prior to in vivo evaluation, for example to explore different spheroid sizes, minimum culture durations for chondroinductive effect, dexamethasone concentrations, and suitable biomaterials to deliver eBMSCs. Longer term, a clinically translational vision may be for dexamethasone-primed, allogeneic eBMSC spheroids to be delivered via an injectable biomaterial capable of supporting weight-bearing and inducing cartilage regeneration in equine cartilage defects.

## Conclusion

The current study provides important insight into the chondroinductive potential of eBMSCs and eUCMSCs in response to several factors and conditions in vitro. Remarkably, neither eBMSCs nor eUCMSCs exhibited a chondroinductive potential with the evaluated chondroinductive compounds and peptides as compared to TGF-β3. Additionally, eUCMSCs showed limited chondrogenic potential under the tested conditions, consistent with previous findings in the equine literature. Nevertheless, eUCMSCs remain a cell source of interest for immunomodulatory potential. Most notably, our findings revealed that dexamethasone, particularly in hypoxic spheroid culture, was a potent and sufficient signal for chondrogenesis of eBMSCs, without the need for the additional growth factor TGF-β3. eBMSCs, under the right culture conditions, are capable of effectively undergoing chondrogenic differentiation, and may represent the most promising candidate for cell-based therapies in equine cartilage regeneration.

## Methods

### Cell culture

#### Umbilical cord tissue and bone marrow harvest

Upon normal parturition, approximately 12 inches from the fetal side or the cord were removed, while the majority of membranes were retained within the mare prior to natural expulsion. The umbilical cords were washed thoroughly with PBS, and then placed into a sterile urine sample cup and submerged in fresh PBS. Seven cords were collected immediately after birth, stored in phosphate-buffered saline (PBS), and shipped overnight cold (not frozen) on ice packs from Colorado State University (CSU) to the University of Oklahoma (OU). Equine UCMSCs were harvested within 48 h of birth. The harvest protocol was adapted from Wang et al. [58] with minor modifications. Once received, the umbilical cords were washed under running water, then moved into a biosafety cabinet, soaked in ethanol for 15 min, and then washed with PBS supplemented with 1% antibiotic-antimycotic (anti-anti, cat# 15240062, ThermoFisher Scientific, Waltham, MA). The blood vessels were then removed, and the umbilical cord matrix (i.e., Wharton’s jelly) was minced into 1–2 mm^3^ pieces and digested in Dulbecco’s Modified Eagle Medium (DMEM, cat# 11885084, ThermoFisher Scientific) supplemented with 0.2% collagenase type II (cat# LS004176, Worthington Biochemical Corporation, Lakewood, NJ) for 16–18 h at 37 °C. Following digestion, the medium was diluted with PBS, centrifuged, and then the supernatant was discarded. The pellet was resuspended in DMEM supplemented with 10% fetal bovine serum (FBS, cat# 16000044, Fisher Scientific) and 1% anti-anti, and then transferred to 75 cm^2^ flasks. Bacterial or fungal contamination was detected at passage 0 or 1 in cells harvested from seven cords and were thus bleached. eUCMSCs from the seven cords were cryopreserved in Recovery™ Cell Culture Freezing Medium (cat# 12648010, ThermoFisher Scientific). Three cords were pooled for the experiments, and donor information are listed in Table 1. eBMSCs were harvested as previously described [14] at CSU. Briefly, bone marrow aspirates were collected using 1000 U/mL of heparin from adult horses, and cells were cultured in DMEM supplemented with 10% FBS, 1% penicillin/streptomycin (P/S, cat# 15140122, ThermoFisher Scientific), and 4-(2-hydroxyethyl)-1-piperazineethanesulfonic acid (HEPES) buffer (cat# 118-089-721EA, Quality Biological, Gaithersburg, MD). eBMSCs were cryopreserved in 95% FBS (cat# SH3091003, FisherScientific, Waltham, MA) and 5% dimethyl sulfoxide (DMSO, ATCC 4-X, Manassas, VA). eBMSCs were shipped from CSU to OU at passage 1 or 2 on dry ice. Six eBMSCs donors were received in total. Donor 1 was a 4-year-old male mixed breed horse, donor 2 was a 2-year-old male Quarter horse, donor 3 was a 3-year-old female Quarter horse, donor 4 was a 2-year-old female mixed breed horse, donor 5 was a 2.5-year-old female mixed breed horse, and donor 6 was a 2.5-year-old gelding (castrated male) mixed breed horse.

#### Cell expansion

Once colonies were established, eBMSC and eUCMSC expansions were done in Minimum Essential Medium alpha (MEM-α, cat# 12561056, ThermoFisher Scientific) supplemented with 10% FBS, 1% anti-anti, 2 ng/mL fibroblast growth factor (FGF, cat# 100-18B, PeproTech) and 25 mM HEPES buffer. Cells were seeded at 3,300 cells/cm^2^ and the medium was changed every 2–3 days. Cells were passaged at 80–90% confluency and used at passage 4. For each cell type, three donors were combined. For eBMSCs, for the experiment evaluating compounds and peptides, cells were used from donors 1, 2, and 3. For the experiment evaluating the effect of dexamethasone, the cells were used from donors 4, 5, and 6. For eUCMSCs, cells from horses A, B, and C were used.

#### Chondrogenic differentiation

Chondrogenic differentiation was induced as previously described [43]. For monolayer culture, cells were seeded at 4,000 cells/well (i.e., 12,500 cells/cm^2^) in a flat-bottom 96-well plates (cat# 62406-081, VWR, Radnor, PA), and for spheroid culture, cells were seeded at 40,000 cells/well in U-bottom 96-well plates (cat# 10861-564, VWR). For spheroid culture, wells were pre-treated with Anti-Adherence Rinsing Solution (cat# 07010, StemCell Technologies, Vancouver, Canada) followed by the centrifugation of the plates at 100 x g for 3 min for pellet formation. Negative control medium was prepared as previously described [43] using DMEM/high glucose/GlutaMAX™ (cat# 10566016, ThermoFisher Scientific), supplemented with 1% P/S, insulin with human transferrin and selenous acid (ITS) + premix 1x (cat# 354352, Corning, Corning, NY), sodium pyruvate 1 mM (cat# 11360070, ThermoFisher Scientific), Minimum Essential Medium (MEM) non-essential amino acids 1x (cat# 11140050, ThermoFisher Scientific), and ascorbate-2-phosphate 50 µg/mL (cat# A8960, Sigma-Aldrich). For the experiment evaluating compounds and peptides, dexamethasone 100 nM (cat# D4902, Sigma-Aldrich) was included in the negative control medium. For the positive control, TGF-β3 10 ng/mL (cat# 8420-B3-005, R&D Systems, Minneapolis, MN) was added to the negative control medium. For the experiment evaluating compounds and peptides, we added KGN (cat# HY-16268, MedChemExpress, Monmouth Junction, NJ) at 1 or 10 µM, SM04690 (cat# HY-109049, MedChemExpress) at 30 or 100 nM, CM10 (Sequence LIANAK, GenScript, Piscataway, NJ) at 100 µM or 200 µM, and/or CK2.1 (Sequence: QIKIWFQNRRKWKKMVPSDPSYEDMGGC, GenScript) at 100 nM or 500 nM. For the experiment evaluating compounds and peptides, cells were incubated at 37 ^o^C in hypoxia (5% O_2_). For the experiment evaluating the effect of dexamethasone, cells were incubated in hypoxia or normoxia.

**Table 1:** eUCMSCs donor information

	Mare breed	Mare age	Stallion breed	Stallion age
Horse A	American Quarter Horse	11 years	American Quarter Horse	5 years
Horse B	American Quarter Horse	19 years	American Quarter Horse	17 years
Horse C	Shire	8 years	Shire	18 years

### Flow cytometry

Equine BMSCs and eUCMSCs were evaluated for the expression of cell surface markers listed in Table 2 using the Cytek Northern Lights Flow Cytometer (Cytek Biosciences, Fremont, CA). Antibodies were selected based on their reported reactivity with equine epitopes by the supplier, or based on the validation of the reactivity of the antibody clone with equine epitopes in previous publications (Table 2) [47, 48]. Briefly, the cultured cells were harvested and suspended at 10^6^ cells/mL. Cells were incubated with conjugated primary antibodies for 25 min at 4 °C. Unstained controls were used as negative controls. Data analysis was performed using the FCS Express 7 (De Novo software, Pasadena, CA). The percentage of positive cells was determined by gating on the negative control.

**Table 2:** Antibodies used for flow cytometric analysis of cell surface markers of eBMSCs and eUCMSCs

Marker	Antibody	Supplier	Part number	References
CD29	Anti-human CD29 Antibody-clone TS2/16-Reacts with horse	BioLegend	303,003	[47]
CD44	Rat anti-mouse-CD44 Antibody-clone IM7-Reacts with horse	Thermo Scientific	MA1-10229	
CD45	Mouse anti-human CD45 antibody-clone F10-89-4-Reacts with horse	Biorad	MCA87A700T	[48]
CD73	Mouse anti-Human/Equine CD73 antibody-clone 606,112	R&D	FAB5795S	
CD79	Mouse anti Human CD79a- clone HM57-Reacts with Horse	Biorad	MCA2538A647T	[47, 48]
CD90	CD90 Monoclonal Antibody-clone 5E10-Reacts with horse	Thermo Scientific	A15726	
CD105	Mouse anti-human CD105 antibody-clone SN6-Reacts with horse	Biorad	MCA1557SBV440	[47]

### Real-time quantitative polymerase chain reaction

The Quick-RNA Miniprep Plus Kit (cat# R1058, Zymo Research, Irvine, CA) was used for the isolation and purification of total RNA on day 21, from cells grown in monolayer and from spheroids as previously described [43]. Afterward, the total RNA was transcribed to cDNA using the High-Capacity cDNA Reverse Transcription kit (cat# 4368813, ThermoFisher Scientific) and real-time quantitative polymerase chain reaction (RT-qPCR) was performed using the TaqMan^®^ Fast Universal PCR Master Mix (cat# 4366073, ThermoFisher Scientific) on the Analytik-Jena QTower^3^-G real-time thermocycler (AnalytikJena, Jena, Germany). The following TaqMan probes were used: GAPDH (Assay ID# Ec03210916_gH), aggrecan (ACAN, Assay ID# Ec03469667_m1), collagen I (COL I, Assay ID# Ec03469676_m1), collagen II (Col2a1, Assay ID# Ec02622868_m1), and SOX-9 (Assay ID# Ec03469763_s1). For spheroid cultures, eight samples from each group (*n* = 8) were tested in duplicate. For monolayer culture, four samples from each group (*n* = 4), with every sample prepared by combining two wells, were tested in duplicate. The ΔΔCt method was used to calculate relative levels of gene expression. GAPDH was used as the endogenous reference gene. For the experiment evaluating compounds and peptides, the negative control group was the calibrator group. For the experiment evaluating the effect of dexamethasone, the control group without dexamethasone and without TGF-β3 was used as the calibrator group.

### Biochemical assays

On day 21, cells were digested in papain solution consisting of 125 mg/mL papain from papaya latex, 5 mM N-acetyl cysteine, and 5 mM EDTA, in PBS. Samples were digested overnight at 60 °C. The DNA content of the samples was measured using a PicoGreen assay kit (cat# P7589, ThermoFisher Scientific) following the manufacturer’s protocol.

Glycosaminoglycan (GAG) content was determined using the dimethylmethylene blue (DMMB) assay. DMMB dye solution (pH = 3) was prepared as previously described [59] and chondroitin sulfate A sodium salt (cat# C9819, Sigma-Aldrich Corporation) was used for standard preparation. For spheroid cultures, eight samples from each group (*n* = 8) were tested in duplicate. For monolayer culture, four samples from each group (*n* = 4), with every sample prepared by combining two wells, were tested in duplicate.

### Statistical analysis

All graphs and statistical analyses were performed using GraphPad Prism 9 (GraphPad Software Inc., La Jolla, CA). Evaluation of quantile-quantile (QQ) plots showed that the data follow a normal distribution. One-way ANOVA or two-way analyses were performed followed by a Tukey post hoc correction. Results were considered significant at *p* < 0.05. Error bars on graphs show the standard deviation of the mean.

## Data Availability

All relevant data is contained within the manuscript. The data generated and analyzed during the current study are available from the corresponding author on request.
